# How Outdoor Trees Affect Indoor Particulate Matter Dispersion: CFD Simulations in a Naturally Ventilated Auditorium

**DOI:** 10.3390/ijerph15122862

**Published:** 2018-12-14

**Authors:** Bo Hong, Hongqiao Qin, Runsheng Jiang, Min Xu, Jiaqi Niu

**Affiliations:** College of Landscape Architecture & Arts, Northwest A&F University, Yangling 712100, China; hongqiao@nwafu.edu.cn (H.Q.); jrs_96@nwafu.edu.cn (R.J.); xuminstudy@nwafu.edu.cn (M.X.); niujq@nwafu.edu.cn (J.N.)

**Keywords:** trees, natural ventilation potential, computational fluid dynamics (CFD), particulate matter (PM_1.0_, PM_2.5_ and PM_10_), indoor air quality, auditorium

## Abstract

This study used computational fluid dynamics (CFD) models, coupling with a standard *k-ε* model based on the Reynolds-averaged Navier-Stokes (RANS) approach and a revised generalized drift flux model, to investigate effects of outdoor trees on indoor PM_1.0_, PM_2.5_, and PM_10_ dispersion in a naturally ventilated auditorium. Crown volume coverage (*CVC*) was introduced to quantify outdoor trees. Simulations were performed on various *CVCs*, oncoming wind velocities and window opening sizes (wall porosities were 3.5 and 7.0%, respectively, for half and fully opened windows). The results were as follows: (1) A vortex formed inside the auditorium in the baseline scenario, and the airflow recirculation created a well-mixed zone with little variation in particle concentrations. There was a noticeable decrease in indoor PM_10_ with the increasing distance from the inlet boundary due to turbulent diffusion. (2) Assuming that pollution sources were diluted through the inlet, average indoor particle concentrations rose exponentially with increasing oncoming wind speed. PM_10_ changed most significantly due to turbulent diffusion and surface deposition reduction intensified by the increased wind velocity. (3) Increasing the window opening improved indoor cross-ventilation, thus reducing indoor particle concentrations. (4) When 2.87 m^3^/m^2^ ≤ *CVC* ≤ 4.73 m^3^/m^2^, indoor PM_2.5_ could meet requirements of the World Health Organization’s air quality guidelines (IT-3) for 24-hour mean concentrations; and (5) average indoor particle concentrations had positive correlations with natural ventilation rates (*R*^2^ = 0.9085, 0.961, 0.9683 for PM_1.0_, PM_2.5_, and PM_10_, respectively, when the wall porosity was 3.5%; *R*^2^ = 0.9158, 0.9734, 0.976 for PM_1.0_, PM_2.5_, and PM_10_, respectively, when the wall porosity was 7.0%).

## 1. Introduction

Rapid urbanization, urban transportation, factory production and domestic biomass combustion have caused a dramatic decline in the quality of urban environments [1,2]. Atmospheric particles such as PM_1.0_, PM_2.5_ and PM_10_ have turned into the most intractable problems of urban air pollution. Epidemiological studies have demonstrated that atmospheric particles are associated with malignancies, respiratory and cardiovascular diseases [3,4,5,6]. As most residents spend 85–90% of their time indoors, indoor air quality is closely related to human health. Therefore, understanding relationships between indoor and outdoor air pollution is critical to better characterize ambient particulate matter exposure and health effects [7,8].

It is well known that vegetation, especially trees, decreases atmospheric particles by capture, filtering, precipitation and turbulent diffusivity [9]. Tree species, canopy porosity, and leaf area density all affect atmospheric particles dispersion [10]. These effects have been recently quantified in wind tunnel tests and experimental studies on deposition velocities and capture efficiency of various trees [11,12,13,14,15]. Relative experimental studies were performed using mass subtraction [16,17], membrane filters [18,19,20,21], the elution-weighed approaches combining particle size analysis [22,23,24,25,26,27], and other direct determination methods [28,29,30]. These studies have largely focused on different particle size distributions of foliage among tree species [31,32], and variation in the particle size distribution on foliage with different dust retention durations [33]. Additionally, the deposition velocities of particulate matter were comparatively analyzed among a variety of trees in numerical simulations [13,34]. The impacts of trees on pollutants are frequently estimated using three-dimensional models, allowing the aerodynamics and deposition effects to be quantitatively calculated [35,36,37,38,39,40,41,42,43,44,45].

Natural ventilation of indoor airflow and cooling is based on outside air movement and pressure differences without mechanical assistance. Previous studies in European cities have shown that using natural ventilation, building energy can be reduced by 40–50% [46,47]. Nevertheless, few studies have examined how to improve indoor air quality through natural ventilation [48]. As concern for relationships between indoor and outdoor air quality has grown, so has the need for indoor and outdoor aerosol measurement [49]. Yet, some research has reported that improper measurement of natural ventilation may aggravate air quality, promoting the need for better indoor air quality measurement [50,51]. Indoor-to-outdoor (I/O) particle concentration ratios, permeability coefficient and other factors have been found to best reflect relationships between indoor and outdoor suspended particulates [52,53,54,55]. Additionally, indoor/outdoor PM_2.5_ has shown a high correlation when there is no obvious indoor particle source [56,57]. Although the impact of outdoor meteorology on indoor particles in naturally ventilated classrooms has been investigated [58], I/O ratios reported have ranged widely from 0 to 5. This is perhaps because the spatial distribution of particles changes noticeably in built environments owing to a range of environmental conditions, particle sizes, building envelopes, greenspace layout and indoor pollution sources [59]. It is clearly difficult to fully understand the links between outdoor and indoor air quality simply based on the I/O ratio.

Numerous experimental studies, wind tunnel experiments and numerical simulations have suggested that plants are effective for abating atmospheric particles. Indoor particulate matter typically originates from outdoor spaces and building ventilation has significant impacts on indoor air pollution [60]. So far, a significant contribution has been made by Tong et al. in uncovering the impact of traffic-related air pollution on indoor air quality of a naturally ventilated building with realistic building characteristics and construction practices, where conditions with a complex internal zone layout were discussed [61]. However, few studies have focused on effects of outdoor trees on indoor/outdoor particles transportation. This study highlights the research gap relating outdoor tree planting on indoor particle concentration distributions. In this aspect, a key issue is to determine how to reduce particulate pollutant transportation from outdoors to indoors by optimizing tree planting and improving natural ventilation.

To fill this research gap, this study used computational fluid dynamics (CFD) models, integrating a standard *k-ε* model based on Reynolds-averaged Navier-Stokes (RANS) approach and a revised generalized drift flux model to predict the spatial relationship between indoor and outdoor pollutants via natural ventilation in a generalized auditorium. Our principle objective is to quantify the impact of outdoor trees on indoor/outdoor relationships of PM_1.0_, PM_2.5_ and PM_10_ concentration distributions with different oncoming wind velocities and window opening sizes. The simulations were organized as follows: First, we described the CFD-based airflow and particle diffusion models. Second, the CFD models were validated by wind tunnel tests and a model chamber with a Phased-array Doppler Anemometry (PDA) experiment. Finally, we evaluated the impact of various parameters on indoor air quality through a series of quantitative simulations.

## 2. Methodology

### 2.1. Simulation Model

In this study, we reproduced airflow and particle diffusion in an idealized auditorium and simulated conditions using three-dimensional steady-state isothermal flow field models. CFD simulations were aligned with the COST Action 732, including choices of target variables, approximation equations, geometrical representation of obstacles, computational domain, boundary conditions, initial data, computational grid, numerical approximations, and iterative convergence criteria [62]. The Reynolds-averaged Navier-Stokes (RANS) model, consisting of the *k-ε* Murakami-Mochida-Kondo (MMK) closure scheme, was used. This model was modified based on the standard *k-ε* model that could better represent airflow fields around buildings. The Semi-Implicit Method for Pressure-Linked Equations (SIMPLE) algorithm with the Quadratic Upstream Interpolation for Convective Kinematics (QUICK) discretization scheme was applied to all governing equations. The scaled iterative convergence criteria for all parameters in simulations were set to 10^−6^. Simulations were run on an i7 2.67 GHz processor (Intel, Santa Clara, CA, USA). The Parabolic Hyperbolic or Elliptic Numerical Integration Code Series (CHAM, London, UK, Edition: 2009) program was used to operate solutions.

In the airflow model, vegetation is parameterized as a porous medium. For calculation efficiency, tree branches and trunks are approximated as foliage and crowns, so that tree canopies (including branches and trunks) are modeled as a whole [63]. From the perspective of aerodynamics, trees reduce air velocity by applying resistance and pressure. Therefore, the resistance was taken into account based on momentum equations to simulate the effects of trees on turbulent flow fields. For extra sources of turbulent flow, the turbulent flow formation on tree canopies and its dissipation were also accounted for. Model equations are as:

Continuity equation:

(1) $$\frac{\partial \langle u_{i}\rangle }{\partial x_{i}}=0$$

where *u_i_* is spatial mean velocity (m/s), and *x_i_* is spatial coordinate.

Momentum equation:

(2) $$\frac{\partial \langle u_{i}\rangle }{\partial t}+\langle u_{j}\rangle \frac{\partial \langle u_{i}\rangle }{\partial x_{j}}=-\frac{1}{\rho }\frac{\partial \langle P\rangle }{\partial x_{i}}+\frac{\partial }{\partial x_{j}}\left(\nu_{eff}\frac{\partial \langle u_{i}\rangle }{\partial x_{j}}\right)+F_{d}$$

where *P* is pressure (Pa); *ρ* is air density (kg/m^3^); *F_d_* is an extra source of resistance on tree canopies; *v_eff_* is effective viscosity (Ns/m^2^), which is calculated as:

(3) $$\nu_{eff}=\nu +\nu_{t}=\nu +c_{\mu }\frac{k^{2}}{\varepsilon }$$

where *k* is turbulent kinetic energy (Nm); *ε* is dissipation rate (m/s); *v_t_* is turbulent viscosity (Ns/m^2^); and *c_μ_* is an empirical coefficient (set to 0.09) [64].

Based on conventional parameterization of plant-airflow interactions, *F_d_* is expressed as:

(4) $$F_{d}=-\frac{1}{2}C_{d}\eta au_{i}S$$

(5) $$S={\left(\sum {\langle u_{i}\rangle }^{2}\right)}^{0.5}$$

where *S* is mean wind velocity (m/s); *C_d_* is a drag coefficient for tree canopies. The drag coefficient for trees is estimated as 0.1 ≤ *C_d_* ≤ 0.3 [65]. In this study, *C_d_* is set to 0.2; *α* is leaf area density (m^2^/m^3^), and *ηα* is the projected area of foliage on the plane perpendicular to the prevailing wind direction (m^2^).

To model turbulence interactions between tree canopies and fluid (air), the kinetic turbulence equation is expressed as:

(6) $$\frac{\partial k}{\partial t}+\langle u_{j}\rangle \frac{\partial k}{\partial x_{j}}=\frac{\partial }{\partial x_{i}}\left(\frac{\nu_{t}}{\sigma_{k}}\frac{\partial k}{\partial x_{i}}\right)+\nu_{t}\left(\frac{\partial \langle u_{i}\rangle }{\partial x_{j}}+\frac{\partial \langle u_{j}\rangle }{\partial x_{i}}\right)\frac{\partial \langle u_{i}\rangle }{\partial x_{j}}-\varepsilon +P_{k}+L_{k}$$

*P_k_* and *L_k_* are extra source terms of the equation, and expressed as:

(7) $$P_{k}=\frac{1}{2}C_{d}\eta aS^{3}$$

(8) $$L_{k}=-2C_{d}\eta aSk$$

Likewise, while considering impacts of tree canopies, the dissipation rate of kinetic energy is calculated as:

(9) $$\frac{\partial \varepsilon }{\partial t}+\langle u_{j}\rangle \frac{\partial \varepsilon }{\partial x_{j}}=\frac{\partial }{\partial x_{i}}\left(\frac{\nu_{t}}{\sigma_{\varepsilon }}\frac{\partial \varepsilon }{\partial x_{i}}\right)+C_{\varepsilon 1}\frac{\varepsilon }{k}\nu_{t}\left(\frac{\partial \langle u_{i}\rangle }{\partial x_{j}}+\frac{\partial \langle u_{j}\rangle }{\partial x_{i}}\right)\frac{\partial \langle u_{i}\rangle }{\partial x_{j}}-C_{\varepsilon 2}\frac{\varepsilon^{2}}{k}+P_{\varepsilon }+L_{\varepsilon }$$

*P_ε_* and *L_ε_* are extra source terms of the above equation, and determined as follows:

(10) $$P_{\varepsilon }=\frac{\varepsilon }{k}C_{p\varepsilon 1}C_{d}\eta aS^{3}$$

(11) $$L_{\varepsilon }=-4C_{p\varepsilon 2}C_{d}\eta aS\varepsilon$$

where *σ_k_*, *σ_ε_*, *C_ε1_* and *C_ε2_* are empirical constants, equal to 1.0, 1.3, 1.44, and 1.92, respectively. The comparisons between field data and simulation results suggest that *C_pε1_* and *C_pε2_* are 1.8 and 0.6, respectively [66].

Foliage reduces atmospheric particles largely by deposition [67]. In Eulerian models, particles are deemed continuums in solving conservation equations between mass and molar concentration. These models are widely used for their accuracy and efficiency. The revised generalized drift flux model, which is a type of revised Eulerian model, takes into account the slippage between particles and the fluid (air). In the model, plants intensify particle deposition via turbulent diffusion. Plants also absorb particles, some of which may re-suspend over foliage [11]. To accommodate these, the deposition and resuspended effects of plants on particulate matter are conveyed through extra terms *S_sink_* and *S_resuspension_*. In this way, the revised generalized drift flux model can simulate an actual built environment comprehensively and accurately [68,69]. It is expressed as:

(12) $$\frac{\partial \left[\left(V_{j}+V_{slip,j}\right)C\right]}{\partial x_{j}}=\frac{\partial }{\partial x_{j}}\left[\varepsilon_{p}\frac{\partial C}{\partial x_{j}}\right]+S_{c}-S_{\sin k}+S_{resuspension}$$

The slippage velocity of particles (*V_slip_*) is defined by gravity, thermal force by the thermophoresis effect, particle fluctuation due to turbulence and particle acceleration [68], and is calculated as:

(13) $$V_{slip,j}=\tau_{p}g_{j}+\tau_{p}\sum F_{j}+\frac{\tau_{p}}{C}S_{mj}-\frac{\tau_{p}}{C}\frac{\partial \left(V_{pj}V_{pi}C\right)}{\partial x_{i}}$$

(14) $$S_{mj}=\frac{\partial }{\partial x_{i}}\left[\varepsilon_{p}C\left(\frac{\partial V_{pj}}{\partial x_{i}}+\frac{\partial V_{pi}}{\partial x_{j}}\right)\right]+\frac{\partial }{\partial x_{i}}\left[\varepsilon_{p}\left(V_{pi}\frac{\partial C}{\partial x_{j}}+V_{pj}\frac{\partial C}{\partial x_{i}}\right)\right]$$

(15) $$\tau_{p}=\frac{C_{c}\rho_{p}d_{p}^{2}}{18\mu }$$

The effect of tree canopies on absorbing atmospheric particles is dependent upon their leaf area densities (LADs), deposition velocities and particulate matter concentrations, conveyed as follows:

*S_sink_*= α(z) × *V_d_* × C (16)

The resuspension of particulate matter, as a term of volume source, is described as:

*S_resuspension_* = *S_sink_* × *P_resuspension_* (17)

*P_resuspension_* = −0.00041*v*^2^ + 0.017*v* − 0.0016 (18)

Since grass is depicted as a plane in this model, the leaf area density of grass is expressed as [40]:

LAD*_grass_* = S/*V* × LAI*_grass_* (19)

where *V_j_* and *V_slip,j_* are the respective mean fluid (air) velocity and gravitational settling velocity of particles in direction *j* (m/s). *C* is particle concentration at the inlet (μg/m^3^); *ε_p_* is turbulent diffusivity (m^2^/s), and can be simplified to 1 [68]. *S_c_* is the formation rate of particle sources (kg/m^3^s). *S_sink_* is the mass of particle absorbed by vegetation per cubic meter within a unit of time (μg/m^3^); *S_resuspension_* is the secondary pollutant generated by foliage per cubic meter within a unit of time [70]; *V_d_* is particle deposition velocity on foliage (m/s); *P_resuspension_* is the percentage of resuspended particles; *v* is the magnitude of air velocity (m/s), and *α* is LAD (m^2^/m^3^). *V_pj_* and *V_pi_* are velocities of particles in directions *j* and *i* (m/s), respectively. *τ_p_* is the relaxation time of a particle; *g_j_* is the gravitational acceleration in direction *j* (m/s^2^); ΣF_j_ is the resultant force exerted upon the particle (m/s^2^); *S_mj_* is the momentum source of particle in direction *j* (kg/(m^2^ s^2^); *μ* is the molecular kinematic viscosity of air(Ns/m^2^); *ρ_p_* is the density of atmospheric particles (kg/m^3^); and *d_p_* is the particle diameter (m); *C_c_* is the Cunningham factor induced by slippage. *S* is the grass surface area (m^2^), *V* is grass volume within the computational domain (m^3^), and LAI*_grass_* is the leaf area index of grass (m^3^/m^3^).

### 2.2. Model Validation

#### 2.2.1. Aerodynamic and Deposition Effects of Trees around Buildings

Ji et al. studied the impact of trees on PM_2.5_ distribution around buildings using wind tunnel experiments [71]. In their tests, buildings and trees were arranged perpendicularly to the oncoming wind at a scale of 1:20 with dimensions of 18L × 12W × 3.5H. The dimension of a single building is 1.5 mL × 0.75 mW × 0.85 mH. To test the impact of trees on airflow and PM_2.5_ distribution more accurately, fresh cypress branches and leaves were used. Cypress was fully leached to remove dust. The crown diameter and tree height were 0.23 and 0.35 m, respectively. A “line source” of particles was released from the inlet with an oncoming wind velocity of 4.5 m/s in the wind tunnel. The airflow field and PM_2.5_ concentration distributions around buildings at the pedestrian height (85 mm), mid-canopy height (325 mm), mid-building height (425 mm), three-quarters of the building height (637.5 mm), and the top of the building (850 mm) in the vertical plane were investigated.

**Figure 1:**
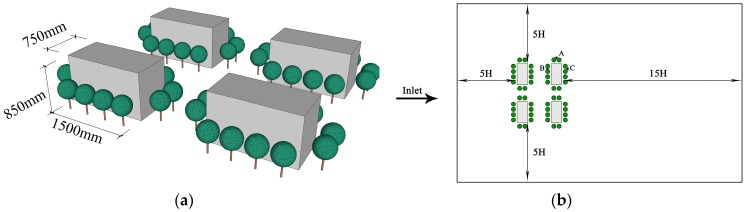
Setup for computational fluid dynamics (CFD) validation. (**a**) The building and tree models were replicated in the wind tunnel test; (**b**) Top view of the computational domain setting.

The simulation model was built based on the size of buildings and trees (Figure 1). Following research guidelines [62,72], the distance between the windward side of front-row buildings and the inflow boundary was set to 5H, while the distance between the leeward side of back-row buildings and the outflow boundary was 15H. The distance between the left/right symmetric boundary and the target area was 5H, and the distance from the ground to the top boundary was 11H. The oncoming wind at the inlet is gradient wind and is conveyed by the equation:

(20) $$u\left(z\right)=u_{0}{\left(z/z_{0}\right)}^{\alpha }$$

where *u*(*z*) is horizontal velocity at height *z*, and *u*_0_ is the horizontal velocity at height *z*_0_. In this model, *u_o_* = 4.5 m/s, *z_o_* = 3.5H m, and *α* = 0.25 [73].

The turbulent kinetic energy, *k* (m^2^/s^2^), and its dissipation rate, *ε* (m^2^/s^3^), are set as:

(21) $$k=\frac{u_{*}^{2}}{\sqrt{C_{\mu }}}\left(1-\frac{z}{\delta }\right)$$

(22) $$\varepsilon =\frac{u_{*}^{3}}{kz}\left(1-\frac{z}{\delta }\right)$$

where u_*_ is friction velocity, *δ* is depth of boundary layer, *k* is von Kàrmàn constant. In this model, u_*_ = 0.52 m/s, *k* = 0.4, and *C_μ_* = 0.09 [74].

In the computational domain, we set the outflow boundary conditions with fixed pressure and zero gradients. Rough wall functions were used for the ground-level boundary. Corresponding constant horizontal velocity and turbulent kinetic energy of the inflow profile were fixed at the top boundary, while the left and right symmetric boundary was modeled as a slippage wall without gradient. To better replicate conditions during the wind tunnel experiment, pollution sources were added to the inlet boundary in simulations.

Three types of structural hexahedral meshes with varying sizes (coarse mesh: *X_min_* = *Y_min_* = *Z_min_* = 0.10H’, fine mesh: *X_min_* = *Y_min_* = *Z_min_* = 0.02H’, and finest mesh: *X_min_* = *Y_min_* = *Z_min_* = 0.01H’) were set in the target area. Calculations were performed using equations (23) to (25) with the grid convergence index (*GCI*) to test grid independence [75]. The *GCI* of coarse and fine meshes was 3.47%, while the *GCI* of fine and finest meshes was 2.97%. Both *GCIs* were below 5%, which suggested that fine meshes were adequate for calculations [76].

(23) $$GCI=F_{s}\frac{\xi_{rms}}{r^{p}-1}$$

(24) $$\xi_{rms}={\left(\frac{\sum_{i=1}^{n}\xi_{i,u}^{2}}{n}\right)}^{\frac{1}{2}}$$

(25) $$\xi_{i,u}=\frac{u_{i,coarse}-u_{i,fine}}{u_{i,fine}}$$

where *F* and *P* are empirical constants set to 3 and 2, respectively. *r* is the ratio between fine and coarse meshes, and *u* is the velocity (m/s).

**Figure 2:**
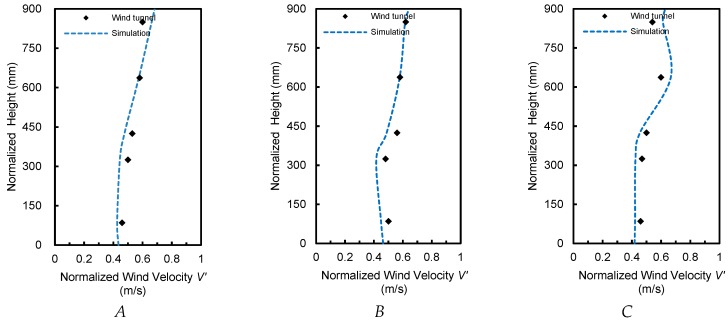
Comparison of normalized wind velocities in simulations and wind tunnel tests at points *A*, *B* and *C*. *V’* = *V_x_*/*V_in_*, *V_x_* is the air speed at points *A*, *B* and *C*, respectively. *V_in_* is the inflow air speed.

**Figure 3:**
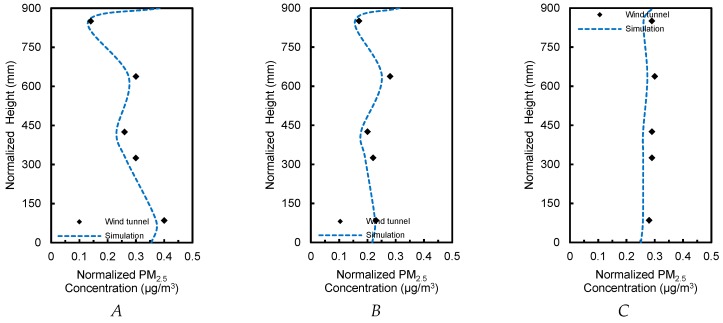
Comparison of normalized PM_2.5_ concentrations in simulations and wind tunnel tests at points *A*, *B* and *C*. *C* = *C_x_*/*C_in_*, *C_x_* is the concentration at points *A*, *B* and *C,* respectively. *C_in_* is the concentration added at the inlet boundary.

We compared normalized wind velocity and PM_2.5_ concentration at different heights at points *A*, *B* and *C* in the simulation and wind tunnel experiment. Mean errors between the simulated and wind tunnel experimental wind velocities were 8.0, 7.4, and 10.3% at points *A*, *B* and *C*, respectively (Figure 2). Mean errors in PM_2.5_ between the simulation and the wind tunnel experiment were 9.1, 8.8, and 9.6% at points *A*, *B* and *C*, respectively (Figure 3). Given that real plant branches and foliage were used in the wind tunnel test, but branch and foliage gaps were ignored in the model (i.e., the crown is a whole), the resistance to wind increased and the deposition velocity increased accordingly in the simulation. As a result, the simulated values were generally low. Thus, all data fit very well within permissible errors regardless of wind velocity or PM_2.5_ concentration, demonstrating that the model can accurately simulate the impact of trees around buildings on particulate matter dispersion.

#### 2.2.2. Particle Transportation in a Ventilated Chamber

**Figure 4:**
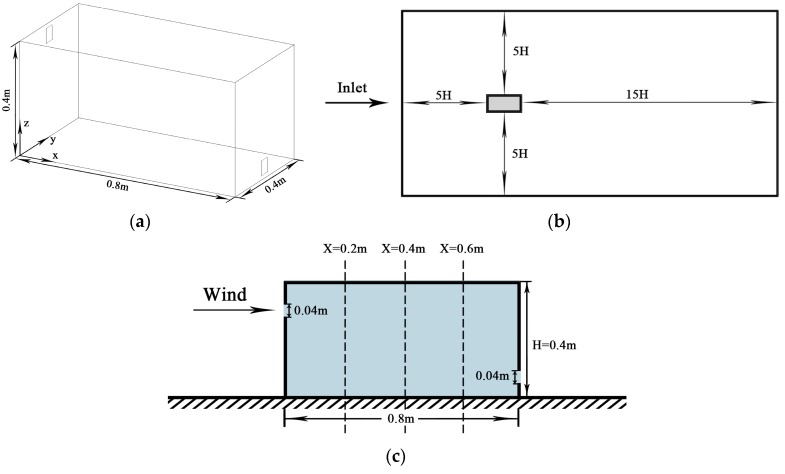
Setup for CFD validation. (**a**) The dimension of the building model replicated in the chamber; (**b**) Top view of the computational domain setting; (**c**) Cross-section along the middle plane (*y* = 0.2 m).

**Figure 5:**
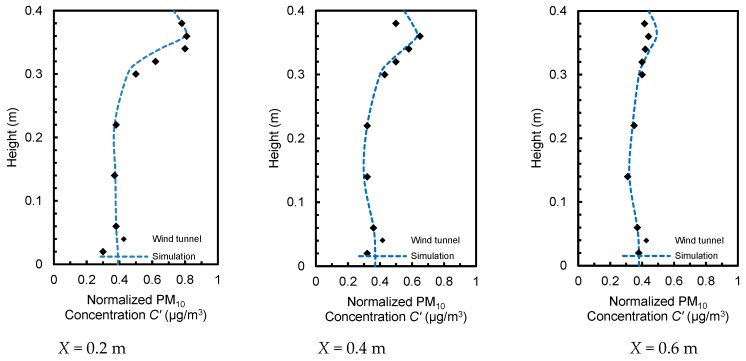
Comparison of normalized PM_10_ concentrations in simulations and experiments at three lines (*x* = 0.2, 0.4, and 0.6 m). *C’* = *C_x_/C_in_*, *C_x_* is the concentration at the three lines, *C_in_* is the concentration added at the inlet boundary.

Chen et al. analyzed the dispersion of particulate matter with a diameter of 10 μm inside a well-ventilated chamber [77]. Figure 4a represents the geometry of the chamber, where the air inlet and outlet (0.04 m × 0.04 m) are aligned with the middle plane (*y* = 0.2 m). In trials, particles were uniformly mixed with supplied air using a separator, and then injected into the chamber with a speed of 0.45 m/s. The model was built in the aforementioned way to calculate the normalized PM_10_ concentration along three lines (*x* = 0.2, 0.4, and 0.6 m) of the central plane and compared with results of an identical chamber of PDA experiments (Figure 4a,c). Mean errors were 8.9, 7.2, and 5.3% on three lines of the central plane, respectively (Figure 5). To determine computational accuracy and cost, the model was parameterized to accurately forecast particle concentration making it feasible to simulate atmospheric particle transportation for the single-sided ventilation using the revised generalized drift flux model.

### 2.3. Simulation Setup

**Figure 6:**
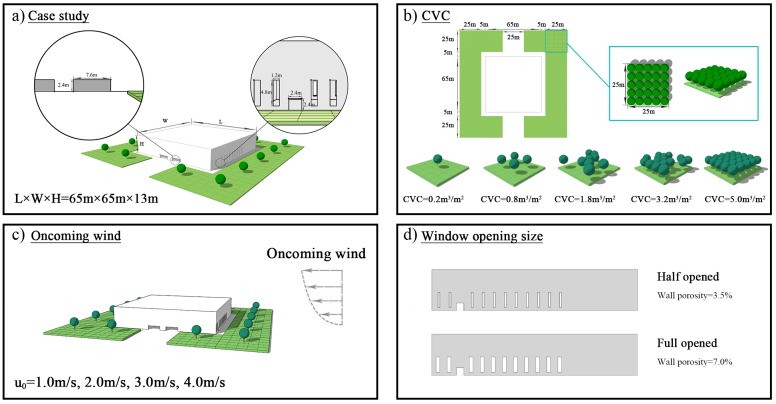
Schematics of simulated test scenarios. (**a**) Dimensions and orientation of test building; (**b**) *CVC* setting; (**c**) Oncoming wind velocity; and (**d**) Window opening size.

We built corresponding building and tree models to analyze the impact of outdoor trees on indoor particulate matter dispersion under natural ventilation. The procedures were quantitatively evaluated by simulating a series of test scenarios using different parameters: tree quantity (*CVCs* = 0.2, 0.8, 1.8, 3.2, and 5.0 m^3^/m^2^), window opening size (wall porosities = 3.5 and 7.0%), and oncoming wind velocity (*u_0_* = 1.0, 2.0, 3.0, and 4.0 m/s) (Figure 6).

The building was a typical auditorium surrounded by green space with an area of 8750 m^2^ in total. The green space was divided into 14 equal units (25 m × 25 m), where trees were planted. The crown volume coverage (*CVC*) was introduced to represent trees planted in the green space and defined as crown volume per area of green space and expressed as [78]:

(26) $$CVC{(m}^{3}{/m}^{2})=\frac{{\mathrm{Total}\;\mathrm{crown}\;\mathrm{volume}\;\mathrm{in}\;\mathrm{green}\;\mathrm{space}\;(m}^{3})}{{\mathrm{Area}\;\mathrm{of}\;\mathrm{green}\;\mathrm{space}\;(m}^{2})}$$

For the simulated auditorium, there were 22 windows (4.8 m × 1.2 m for fully opened windows) on walls and 2 safety exits (2.4 m × 2.4 m) on both sides. The main entrance, front door is 7.6 m × 2.4 m. Cypress (*Platycladus orientalis*), a type of evergreen conifer, was selected in the model. The leaf area density, tree height, crown diameter and crown base height are 2.2 m^2^/m^3^, 5.0 m, 5.0 m and 2.0 m, respectively. The deposition velocities of cypress for PM_1.0_, PM_2.5_ and PM_10_ are set to 0.1949, 0.0371 and 0.0615 m/s, respectively [13,71]. According to the literature [37,40], the leaf area index and height of grass are 2.0 m^3^/m^3^ and 0.5 m, respectively. Additionally, the deposition velocities of grass for PM_1.0_, PM_2.5_ and PM_10_ are 0.02, 0.0028 and 0.0064 m/s, respectively.

**Figure 7:**
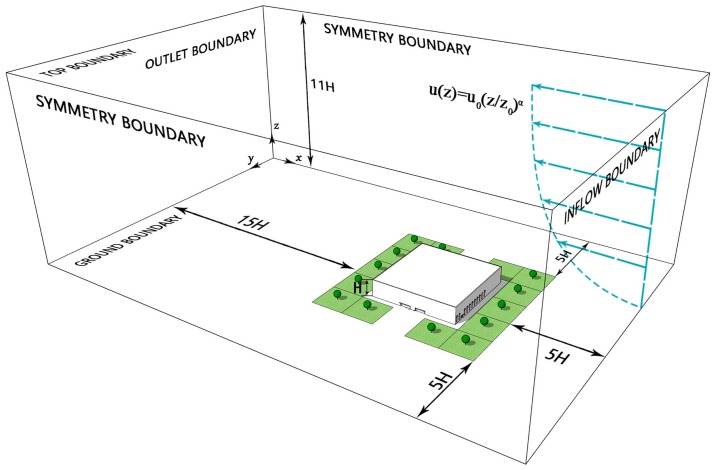
The computational domain.

The computational domain, with a dimension of 385 m × 255 m × 143 m, was divided into three types of structural hexahedral meshes (coarse meshes: *X_min_ = Y_min_ = Z_min_* = 0.10H; fine meshes: *X_min_ = Y_min_ = Z_min_* = 0.04H; and finest meshes: *X_min_ = Y_min_ = Z_min_* = 0.02H) (Figure 7). After grid independence testing, fine mesh (*X_min_ = Y_min_ = Z_min_* = 0.04H) with a total of 3.1 million cells was selected to ensure a higher resolution in the target region. The boundary setting was similar to that described in Section 2.2.1. The pollution sources were assumed to be transported pollutants diluted through the inlet boundary with no other pollution sources in the domain. In addition, infiltration of building walls was not accounted for in the model because natural ventilation flow rates at window openings were far stronger than those from infiltration. Other boundary conditions settings are shown in Table 1.

To further analyze relationships between natural ventilation potential and indoor particle concentrations, the natural ventilation rate was selected as an evaluation index. According to previous research [81,82], wind pressure is the main driving force of natural ventilation in the study area. Airflow penetrates buildings and trees as a consequence of pressure differences generated on building facades. Affected by wind deflection, the windward wind pressure of a building facade is generally positive, whereas the leeward is negative. Thus, the wind pressure on the facade of the building is expressed as:

*P_w_* = 0.5*C_p_ρV*^2^ (27)

where *P_w_* is wind pressure (Pa), *C_p_* is a static pressure coefficient, *ρ* is air density (kg/m^3^), and *V* is wind velocity (m/s).

The natural ventilation rate is expressed as follows:

(28) $$G=F_{j}\sqrt{\frac{2\left|\Delta P\right|\cdot \rho }{\left[{\left(\frac{F_{j}}{F_{p}}\right)}^{2}\frac{\zeta_{p}}{\zeta_{j}}+1\right]\cdot \zeta_{j}}}$$

where *G* is the natural ventilation rate (kg/s); *F_j_* and *F_p_* are the inlet and outlet area (m^2^), respectively; *ζ_j_* and *ζ_p_* are coefficients of local resistance at the inlet and outlet, set as 2.59 and 8, respectively [83]. |∆*P*| is the difference in wind pressure on the facade of windward and leeward sides of the buildings (Pa), and *ρ* is air density (kg/m^3^).

## 3. Results and Discussion

### 3.1. Baseline Scenario

**Figure 8:**
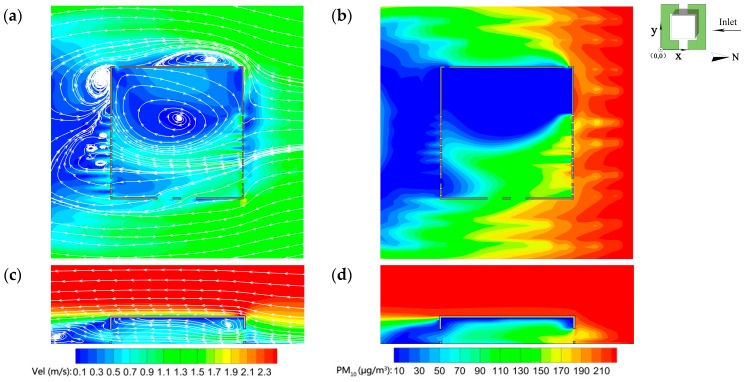
Airflow fields at *z* = 1.5 m (**a**), PM_10_ distributions at *z* = 1.5 m (**b**), airflow fields at *y* = 53 m (**c**), and PM_10_ distributions at *y* = 53 m (**d**) in the baseline scenario.

Figure 8 depicts the contours of airflow fields and PM_10_ distributions in the baseline scenario (oncoming wind velocity = 2.0 m/s, *CVC* = 0.8 m^3^/m^2^, wall porosity = 3.5%). This demonstrated that the wind velocity increases dramatically at the turn of the windward wall. Airflows penetrate inwards from the north of the auditorium. The wind velocity slows gradually and reaches a minimum near the windward wall. An intense vortex forms in the west of the auditorium, where the wind velocity is relatively low. There are four small vortices and a relatively large vortex at the south of the auditorium. Inside the auditorium, airflows penetrate through areas where windows are open. This forms long and narrow ventilation corridors where wind velocity is relatively high. At the east of these ventilation corridors, the wind velocity is variable. A large vortex forms in the west of the auditorium, where the wind velocity is the lowest and uniform (Figure 8a). The windward wind velocity increases progressively with height and rises dramatically when airflows approach the roof (Figure 8c). Similar airflow patterns can be seen in an isolated room with perpendicular wind direction [84]. The windward airflow is separated at the front corner of the building, spreads around the top surface of the building and extends downwind. The wind velocity drops gradually with height and a small clockwise vortex forms near and above the windward wall in the indoor perpendicular direction. Airflows converge near the open windows on the leeward façade where the wind velocity is relatively high. Additionally, a counterclockwise vortex is formed in the turbulent wake area due to the aerodynamic and obstacle effects of trees on airflows.

Trees are effective in reducing atmospheric particles. PM_10_ on both sides of the auditorium is higher than indoors with lanceolate distributions (Figure 8b). The indoor concentration is higher in wind corridors, ranging from 70 to 190 μg/m^3^. Re-circulation inside the auditorium creates a well-mixed zone with little variation in particle concentration. PM_10_ is lower on windward tree canopies owing to the dust-retention ability of trees (Figure 8d). Due to gravitational deposition effects, the indoor particle concentration on the top of the space is lower than that near the ground, which is in accordance with the results of Jin et al. [84].

**Figure 9:**
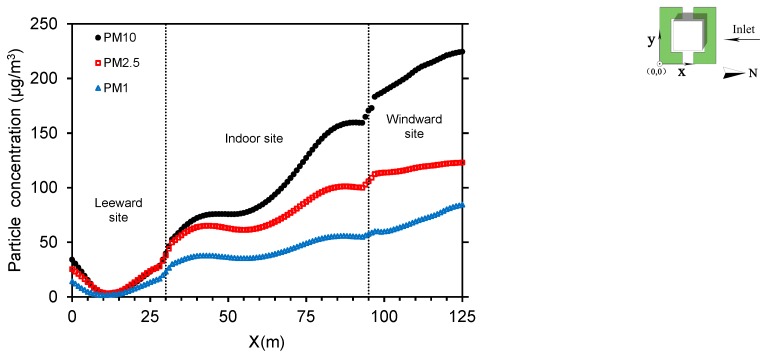
Particle concentrations along the horizontal plane at *y* = 53 m and *z* = 1.5 m in the baseline scenario.

Particle concentrations differ in windward areas, where the longer the distance, the lower the concentrations (Figure 9). A relatively small concentration peak appears near the windward wall of the auditorium. Because airflows converge by windows, where the particles are blocked by walls, there is a slight increase in particle concentrations. Particle concentrations inside the auditorium decrease further from the windward side. In addition, the indoor concentration is 44 to 60% of that at the inlet values due to dust retention effects of plants and windows hindering particle diffusion towards the building interior. Due to turbulent diffusion, PM_10_ declines most significantly, and the deposition rate is relatively high. However, at leeward sites, concentrations are all below 50 μg/m^3^ with smaller differences, regardless of particle size. Concentrations decline faster due to the deposition effect of trees around the leeward sites.

### 3.2. Oncoming Wind Speed and Window Opening Size

**Figure 10:**
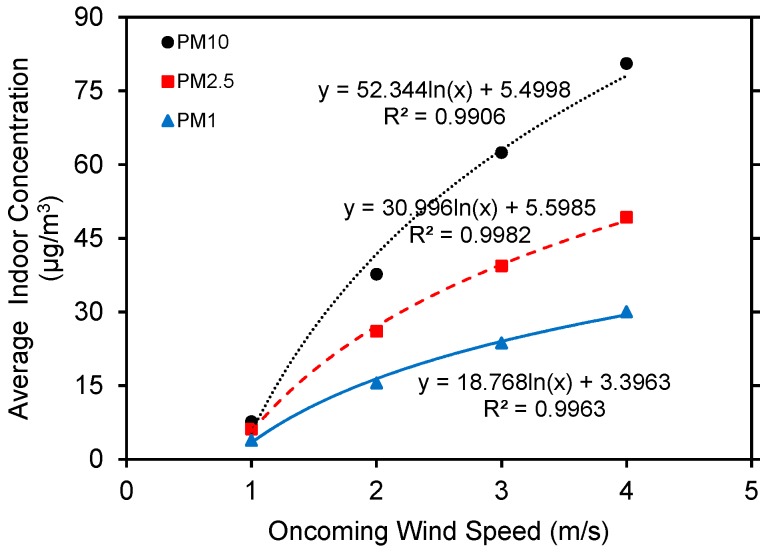
Relationship between oncoming wind speed and average indoor particle concentrations (wall porosity = 3.5%).

Oncoming wind velocities are set to 1.0, 2.0, 3.0, and 4.0 m/s to explore the effects of oncoming wind velocity on indoor particle concentration distributions (Figure 10). For clarity, only scenarios where *CVC* = 0.8 m^3^/m^2^ are presented. Particle concentrations tend to increase exponentially with the increasing oncoming wind velocity whether windows are half or fully open. This appears to because the increased wind velocity enhances indoor/outdoor cross ventilation and particle diffusion. This result is contrary to research indicating a negative correlation between normalized oncoming wind speed and average indoor particle concentrations [61]. The reason is that polluted sources in their study originated from traffic, while our study assumed that the pollution sources were wind transported and diluted through the inlet. Among wind velocity increments, average indoor concentrations differ more significantly among PM_1.0_, PM_2.5_ and PM_10_.These differences are slight in PM_10_, PM_2.5_ and PM_1.0_ when wind velocity is 1.0 m/s. However, PM_10_ and PM_2.5_ differ by 35 μg/m^3^ when the wind velocity reaches 4.0 m/s. PM_10_ varies most significantly with wind velocity, followed by PM_2.5_ and PM_1.0_ successively, as increasing wind velocity facilitates turbulent diffusion of larger particles and diminishes surface deposition.

**Figure 11:**
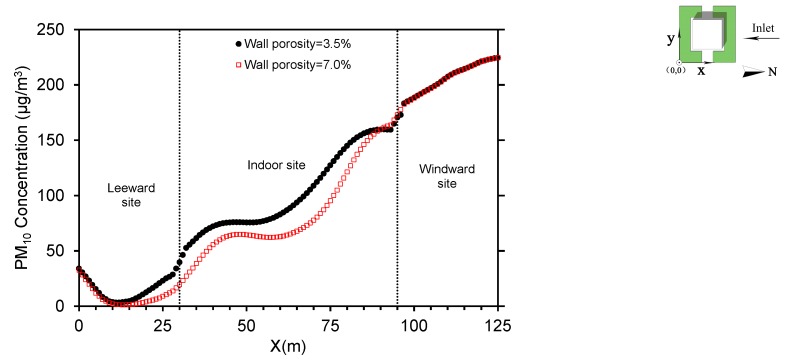
Comparison of indoor and outdoor PM_10_ when wall porosities are 3.5 and 7.0% along the horizontal plane at *y* = 53 m and *z* = 1.5 m.

This section focuses on the effect of window opening size on indoor particle concentrations. For clarity, indoor and outdoor PM_10_ is compared when windows are fully open (wall porosity = 7.0%) and half open (wall porosity = 3.5%) in the baseline scenario (Figure 11). PM_10_ is similar on the windward facade of the auditorium when windows are half or fully open. The airflow converges in relatively small areas and the wind velocity increases when the wall porosity is 3.5%, the turbulent diffusion accelerates as a result. Hence, the indoor PM_10_ is higher than that when the wall porosity is 7.0%. This result agrees well with the study demonstrating that 9% wall porosity resulted in greater normalized concentrations compared with 18% [61]. The difference reaches its maximum when windows are half and fully open within 70 m ≤ *X* ≤ 75 m, at approximately 30 μg/m^3^. This means an increment of 20% in PM_10_ results by closing the windows by half. Near leeward windows, the concentration drops faster when the wall porosity is 3.5%. At leeward sites, the difference decreases gradually and the concentration is approximately the same when 0 m < *X* < 12.5 m, whether the wall porosity is 3.5% or 7.0%.

### 3.3. Crown Volume Coverage

**Figure 12:**
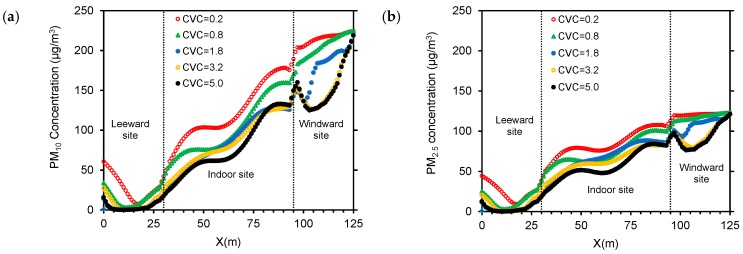

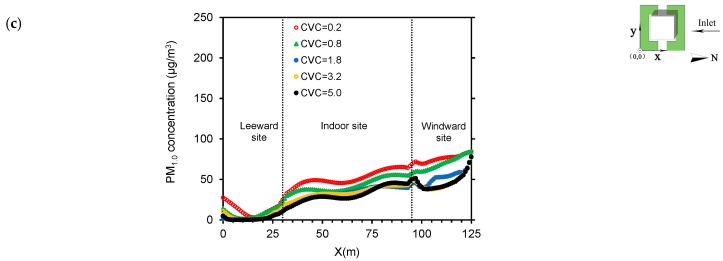
PM_10_ (**a**), PM_2.5_ (**b**) and PM_1.0_ (**c**) along the horizontal plane at *z* = 1.5 m and *y* = 53 m (wall porosity = 3.5%).

Particle concentrations decline gradually with increasing *CVC*, and PM_10_ varies within the widest range (Figure 12). At windward sites, concentrations decline gradually as the distance to the inlet boundary increases. This decrease is most striking when *CVCs* equal 3.2 and 5.0 m^3^/m^2^. Concentrations increase and reach a peak at about 10 m from windward windows. The higher the *CVC*, the more evident the peak. Wind velocity and airflow are lower when *CVC* is higher. Thus, natural ventilation is affected, and cross ventilation becomes weaker, resulting in an increase in particle concentration. Inside the auditorium, the difference of PM_10_ approaches 50 μg/m^3^ when *CVCs* are 0.2 and 5.0 m^3^/m^2^ (Figure 12a). The difference of PM_1.0_ changes consistently with distance. When *CVCs* are 1.8, 3.2 and 5.0 m^3^/m^2^, the concentration difference is relatively small along the horizontal plane (Figure 12c). At leeward sites, particle concentrations decline initially and then increase gradually as the distance from the auditorium increases, and concentration changes tend to be consistent irrespective of *CVC*.

**Figure 13:**
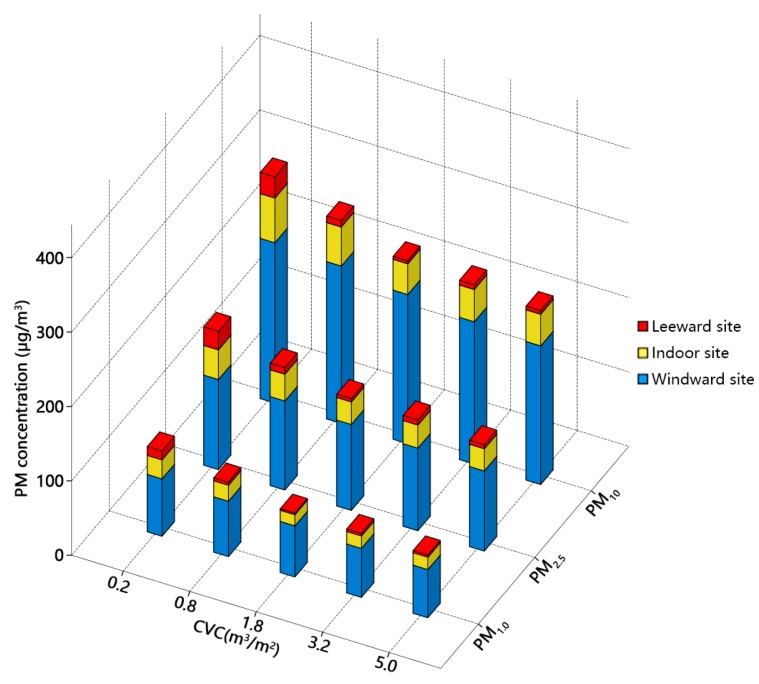
Comparison of PM_10_, PM_2.5_ and PM_1.0_ at windward, indoor and leeward sites at pedestrian level (1.5 m height) among various crown volume coverage (*CVC*) scenarios (oncoming wind velocity = 2.0 m/s, and wall porosity = 3.5%).

Average particle concentrations at pedestrian level (1.5 m height) in windward, indoor and leeward sites among various *CVC*s with oncoming wind velocity of 2.0 m/s and wall porosity of 3.5% scenarios are shown in Figure 13. Concentration distributions clearly differ between windward and leeward sites. Trees planted in windward and leeward sites intercepted particles and reduced the kinetic energy of airflow resulting from the shelter effect of tree canopies. Hence, particulate dispersion was impeded by trees in windward and leeward sites. The concentrations decrease with the increase of *CVC*s. At windward sites, concentrations clearly decline with increasing *CVCs*. The indoor and leeward sites have the smallest particle concentrations when *CVC* is 1.8 m^3^/m^2^. It is also evident that PM_10_ differences among various *CVC*s are larger than those of PM_1.0_ and PM_2.5_. Moreover, with *CVC* increasing, the declining tendency of concentrations became lower when *CVC*s > 1.8 m^3^/m^2^.

**Figure 14:**
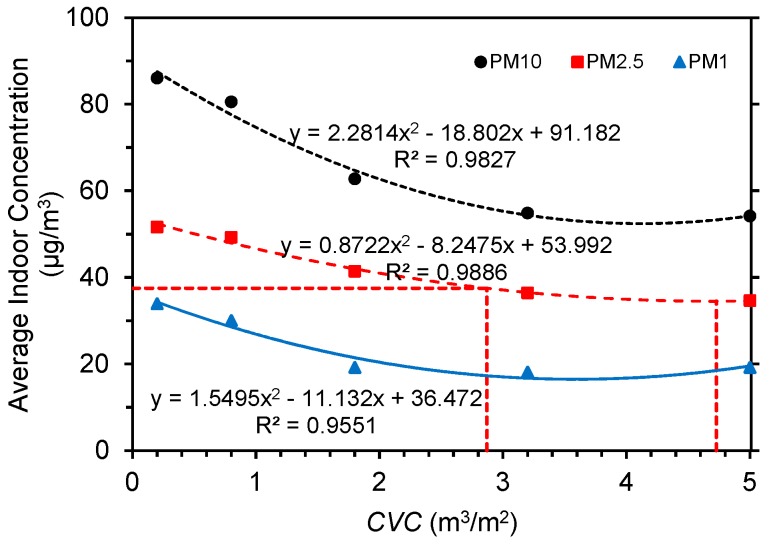
Relationships between *CVC* and average indoor particle concentrations (oncoming wind speed = 4.0 m/s, and wall porosity = 3.5%).

Tree planting density imposes relatively significant impacts on particle concentration distributions [85]. These observations show that the oncoming wind velocity is positively correlated with indoor particle concentration, prompting us to examine connections between *CVC* and indoor concentration in scenarios when the oncoming wind velocity is 4.0 m/s and wall porosity is 3.5% (Figure 14). Particle concentration and *CVC* exhibit significant quadratic patterns. Concentrations decline gradually with *CVC* initially and then increase slowly. This tendency is similar to the results of Yin et al. [78]. PM_10_, PM_2.5_ and PM_1.0_ reach their minima (52.4 μg/m^3^ for PM_10_, 34.5 μg/m^3^ for PM_2.5_, and 16.5 μg/m^3^ for PM_1.0_) when *CVCs* are 4.12, 4.73, and 3.59 m^3^/m^2^, respectively. Average indoor PM_10_ concentration fluctuates most significantly with *CVC*, while PM_2.5_ and PM_1.0_ change slowly, because larger sized particles are more readily deposited on leaves [86].

The World Health Organization air quality guidelines (WHO AQGs) identify PM_2.5_ as an indicator for particulate matter evaluation. We selected interim target-3 (IT-3) with a PM_2.5_ of 37.5 μg/m^3^ as a criterion [87]. When *CVC* > 2.87 m^3^/m^2^, PM_2.5_ concentration is below 37.5 μg/m^3^ and achieves the IT-3 of WHO AQG for 24-hour mean concentrations. Above all, the indoor PM_2.5_ is in line with WHO AQG specifications when 2.87 m^3^/m^2^ ≤ *CVC* ≤ 4.73 m^3^/m^2^.

### 3.4. Relationship Between Indoor Particle Concentrations and Natural Ventilation Rate (G)

**Figure 15:**
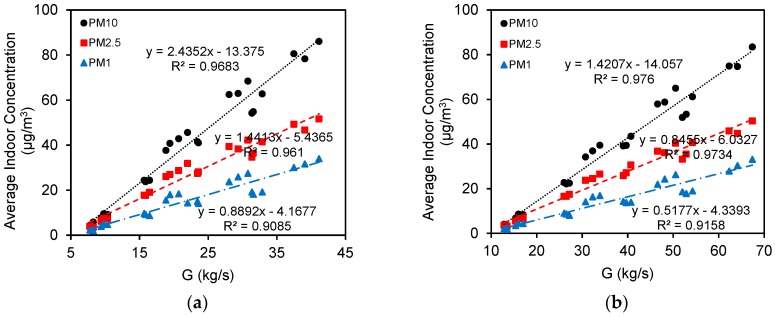
Relationships between *G* and average indoor particle concentrations when wall porosities are 3.5% (**a**) and 7.0% (**b**), respectively.

The relationship between indoor average particle concentration and natural ventilation rate (G) varied among the simulations (Figure 15). Average indoor concentration and natural ventilation rate show a similar trend and a positive correlation. PM_10_ changes most significantly with the natural ventilation rate. Indoor particle concentration clearly increases when the wall porosity is 3.5% compared with that when the wall porosity is 7.0%, because the natural ventilation rate is higher when windows are fully open. When the air exchange frequency is constant, airflow discharges rapidly, removing pollutants and reducing indoor particulate matter. Previous studies have demonstrated that absolute or relative difference in wind pressure in buildings is weakly correlated with indoor particle concentrations [58,88]. Our simulation results indicate that the natural ventilation rate is significantly and positively correlated with indoor particle concentration linked to window opening size. Previous research has also shown that indoor and outdoor particulate concentrations are closely correlated (*R*^2^ = 0.9104) when windows are closed and there are no obvious indoor pollution sources. The indoor/outdoor concentrations ratios (I/O ratios) were significantly correlated with outdoor wind velocity and relative humidity [56]. Our results were based on having the windows open (half open), thus expanding the understanding of relationships on indoor/outdoor particulate matter transportation.

In this study, the impact of outdoor trees on PM_1.0_, PM_2.5_ and PM_10_ inside a typical auditorium was analyzed using numerical simulations. Like the I/O ratio, this CFD method may be used as an alternative approach to investigate relationships between indoor and outdoor particle concentration distributions, especially when trees are planted outside. For future consideration, our study points to some simulation improvements. First, green spaces, by definition, have trees, shrubs and/or herbs. Our study only modeled the impact of trees and herbs, leaving open the question of more diverse green space configurations. Further, this study used *CVC* to quantify the trees outside, which does not reflect the distribution pattern or arrangement of trees. This may exert a different influence on the indoor particulate matter dispersion. Future simulations should take into account the coupling effects of tree distribution patterns on microclimate. Second, airflow is variable and affects particulate diffusion even it is constant, but our simulations set the particles as a steady state without affecting airflow. Hence, our conclusions remain to be validated by more field experiments. Third, the impact of plants on dust retention changes with growth, suggesting that dynamic vegetation growth should be included in future simulations. Fourth, previous research has shown that temperature and solar radiation were weakly correlated with indoor particulate matter (*R*^2^ = 0.32–0.47 for temperature and *R*^2^ = 0.06–0.15 for solar radiation) [55]. However, the effects of solar radiation, and heat transfer between buildings and plants were not taken into account in this simulation. Nevertheless, these factors should be considered in future studies, so that simulation results can better reflect actual conditions. Finally, we did not consider indoor generated particle sources in our simulations. We exclude them because our focus was on the effect of outdoor trees on indoor particle concentration distributions, but in the real built environment, indoor air pollution is affected not only by outdoor pollutants, but also indoor-generated sources, i.e., cooking, smoking, cleaning, and general activity. A comprehensive analysis concerning outdoor/indoor polluted sources, particle penetration and infiltration should be conducted to thoroughly understand outdoor and indoor particle transportation in built environments.

## 4. Conclusions

We adapted computational fluid dynamics (CFD) models, considering the aerodynamic and deposition effect of trees, to investigate the impact of outdoor trees on indoor PM_1.0_, PM_2.5_ and PM_10_ concentration distributions. Using *CVC* as an evaluation index for outdoor tree planting, our study thoroughly compared indoor particle concentration distributions with different *CVCs*, oncoming wind velocities, window opening sizes, and the capacity of natural ventilation potential. The following conclusions can be drawn:

(1) The building envelope restricts airflow and particle dispersion and dilution. In our baseline scenario, a relatively large vortex formed inside the auditorium. As a consequence, the inside air re-circulated and created a well-mixed zone with little variation in particle concentration. Inside the auditorium, the concentration declined with increased distance from the windward side. In addition, the concentration inside was 44 to 60% of that at the inlet boundary, because the dust-retaining ability of trees and window area obstructed diffusion of particles to indoors. Indoor PM_10_ fluctuated most significantly with increasing distance from the inlet boundary. PM_2.5_ and PM_1.0_ changes were not clear because the deposition was more effective among larger particles due to turbulent diffusion.
(2) Under the assumption that pollution sources were diluted through the inlet, the average indoor particle concentration rose exponentially with increasing oncoming wind speed. The difference among PM_1.0_, PM_2.5_ and PM_10_ was relatively small when wind velocity was 1.0 m/s, but the concentration changed significantly as the wind velocity increased to 2.0 m/s. As increased wind velocity intensified turbulent diffusion of larger particles and reduced surface deposition, PM_10_ changed most significantly, followed by PM_2.5_ and PM_1.0_.
(3) Indoor cross-ventilation was improved when the wall porosity was 7.0%. Thus a 20% increment in PM_10_ was achieved by closing windows by half. Near the leeward windows, the concentration declined much more quickly when the wall porosity was 3.5% than that when the wall porosity was 7.0%. The difference gradually narrowed as the distance to the leeward wall increased and the concentration was very similar under the condition when 0 m < *X* < 12.5 m.
(4) The average indoor particle concentration declined initially and then increased with a higher *CVC*. When the *CVC* ranged between 2.87 and 4.73 m^2^/m^3^, the indoor PM_2.5_ concentration could meet the requirement of IT-3 of the WHO AQGs for 24-hour mean concentrations.
(5) Average indoor concentrations were positively correlated with natural ventilation rates and increased more quickly when the wall porosity was 3.5%. Airflow removed more pollutants and shortened longevity of particles indoors when the wall porosity was 7.0%. Thus, the indoor particle concentration was lower than that when the wall porosity was 3.5%.

Our findings indicate that trees planted around buildings, window opening size, and predominate wind velocity all have significant impacts on indoor atmospheric particle concentrations. To plan new buildings, it will be necessary to deliberately evaluate tree planting designs and ambient wind conditions around buildings at early design stages. Such planning will maximize the natural ventilation potential while decreasing the indoor particle concentration. Further, ventilation strategies (changing window opening size), outdoor movable tree planting pools, and layout of interior space may be adapted for existing buildings, so as to lower the indoor particle concentrations. Our conclusions could be used as guidelines for assisting building architects, urban planners and landscape architects to evaluate natural ventilation strategies with enhanced consideration for improving indoor air quality in public buildings.

## Figures and Tables

**Table 1:** Boundary conditions setting *.

Parameter	Definition	Values
Location	Xi’an (China)	latitude: 34.26° N, longitude: 108.07° E
Meteorological conditions	wind speed at the height of 10 m	1.0, 2.0, 3.0, 4.0 m/s
	wind direction	North
Pollution source	PM_1.0_	85 μg/m^3^
	PM_2.5_	123 μg/m^3^
	PM_10_	225 μg/m^3^

* Since the highest air pollution level stretches from mid-November to December, average atmospheric particle concentrations and wind conditions (wind velocities and predominate wind direction) during this time period from the Xi’an statistical yearbook (2017) were selected as boundary parameters [79,80].
